# The Role of Social Relational Emotions for Human-Nature Connectedness

**DOI:** 10.3389/fpsyg.2019.02759

**Published:** 2019-12-17

**Authors:** Evi Petersen, Alan Page Fiske, Thomas W. Schubert

**Affiliations:** ^1^Department of Sports, Physical Education and Outdoor Studies, University of South-Eastern Norway, Bø i Telemark, Norway; ^2^Department of Anthropology, University of California, Los Angeles, Los Angeles, CA, United States; ^3^Department of Psychology, University of Oslo, Oslo, Norway

**Keywords:** connectedness to nature, social relational emotions, social connectedness, kama muta, awe, well-being, sustainability, human-nature relationship

## Abstract

Little is known about the psychological processes through which people connect to nature. From social psychology, we know that emotions play an essential role when connecting to others. In this article, we argue that social connectedness and connectedness to nature are underpinned by the same emotions. More specifically, we propose that *social relational emotions* are crucial to understanding the process through which humans connect to nature. Beside other emotions, kama muta (Sanskrit: being moved by love) might play a particular crucial role when connecting to nature. Future research should consider the role of social relational emotions in human-nature relationships.

“*When one tugs at a single thing in nature, he finds it attached to the rest of the world*”

– John Muir

## Introduction

A positive relationship between humans and the natural world has been shown to be cross-culturally essential to sustain both human well-being and the well-being of the environment. This claim is supported by evidence of health-related and emotional well-being benefits from human interaction with nature (Hartig et al., 2014; McMahan and Estes, 2015) as well as its effects on pro-environmental attitude and behavior to address environmental sustainability issues (Geng et al., 2015; Ives et al., 2018; Rosa et al., 2018). In this context, a substantial body of literature has explored the human relationship and orientation toward nature. Among the conceptualizations are *nature relatedness* (Nisbet et al., 2009), *inclusion of nature in self* (Schultz, 2002), *emotional affinity toward nature* (Kals et al., 1999), and *connectedness to nature* (Mayer and Frantz, 2004). Some of these concepts tap into cognitive appreciation of being embedded in nature, while others focus on the emotional attachment or address material dependence on nature (Ives et al., 2017). Despite their differences, all of the concepts seem to agree on same core phenomenon: a relatively permanent connection to nature on the individual level (Tam, 2013; Capaldi et al., 2014). Psychometric scales used to measure this, such as the *nature relatedness scale* (Nisbet et al., 2009) or the *connectedness to nature scale* (Mayer and Frantz, 2004), are all highly correlated with one another (Tam, 2013). Accordingly, we will not distinguish between the different concepts in this paper, and we will refer to *nature connectedness* as an umbrella term.

Nature connectedness has recently been shown to be a predictor of human well-being (Capaldi et al., 2014), as well as pro-environmental attitudes and behavior (Mackay and Schmitt, 2019). Explanations addressing the general question of why humans connect to nature are for the most part theoretically rooted in the Biophilia Hypothesis. First coined as a term by Fromm (1964) and later expanded by Wilson (1984), the Biophilia Hypothesis originates from the evolutionary notion that humans depend on their natural environment. It is claimed that this dependence evolved into a predisposition to be cognitively and emotionally attracted to nature and to affiliate with it (Kellert and Wilson, 1993). In this sense, the Biophilia Hypothesis provides a basis for an interdisciplinary research agenda to understand the general motivation of humans to connect to nature. However, it leaves the question open how such feelings of connectedness to nature are instantiated.

## Knowledge Gap and Aim of the Paper

We need to understand and investigate the specific pathways that lead to nature connectedness in order to provide possibilities for it to occur. In this scope, psychological mechanisms have been stated to be central when examining the routes to nature connectedness (Zylstra et al., 2014; Lumber et al., 2017). Research from social psychology demonstrates that connecting to others is closely tied to emotional processes (Fiske et al., 2019), but little attention has been given to the emotional mechanisms that presumable enable and mediate connecting to nature (Milton, 2002). Consequently, the present paper adds explanatory value to understanding human-nature interaction by stressing that specific emotions play an important role for connectedness to nature, not simply a side-effect or outcome. To the best of our knowledge, this is the first paper to suggest that social relational emotions have an important role to play in the process of connecting to nature.

In the next sections, we first discuss the relationship between social connectedness and connectedness to nature and explain why both concepts are most likely underpinned by the same emotional mechanisms. We then describe the function of emotions for the process of connecting to social others and highlight the potential of social relational emotions in this scope. In the third paragraph of the paper, we present specific research examples on the role and impact of these emotions in the scope of connecting to nature. We conclude by addressing the significance and implications of social relational emotions for future research.

## Connectedness to Nature and Social Connectedness

Humans are social beings and therefore have a fundamental need to relate (Baumeister and Leary, 1995; Dunbar and Shultz, 2007; Fiske, 2018). This need is often satisfied by socially connecting to others such as the partner, family or friends. However, we know that people also socially relate to animals, deceased ancestors, deities, abstract entities such as countries, humanity as a whole, or even imagined collectivities in order to meet their need to relate (Fiske, 2004; McFarland et al., 2012). Likewise, ecopsychologists have pointed out that the need to relate can be satisfied by feeling connected to nature (Schultz, 2002; Baxter and Pelletier, 2019).

While social psychology has mainly focused on connections between humans, ecopsychology has tried to understand the connection between humans and the natural world. Although little research has specifically investigated the relationship of these two concepts, the literature points to the conceptual similarities between social connectedness and connectedness to nature. First, Kals et al. (1999) and Mayer et al. (2009) demonstrated that like human connections with other humans, positive experiences with nature can lead to an emotional affinity and cognitive identification with nature. Second, both relationships have similar basic features such as commitment (Davis et al., 2009) and inclusion of the other (nature or other human beings) in the self-concept (Schultz, 2002). Third, several studies have found relations between measures of social connectedness and connectedness to nature. For instance, Howell et al. (2011) and Howell et al. (2013) found significant positive correlations between *social connectedness*, framed as social well-being (Keyes, 1998) and the *connectedness to nature scale* by Mayer and Frantz (2004), the n*ature relatedness scale* by Nisbet et al. (2009), and the *allo-inclusive identity nature subscale* by Leary et al. (2008). Finally, the notion of *being connected* itself is a psychological one, which is mediated by culture, context, and experiences (Mesquita et al., 2016). Therefore, it is plausible to hypothesize that connecting to other human beings and connecting to nature are underpinned by the same general psychological mechanisms, which include cognitive, emotional, and behavioral processes. We focus here on specific emotions that play an essential role in connecting to others.

## Social Relational Emotions – Their Function for Social Connectedness

In this paragraph, we discuss what kind of emotions facilitate social connectedness and how. Emotions, in general, play a crucial role in our daily life as they influence how we think and behave (Ekman and Davidson, 1994; Scherer and Ekman, 2009). Emotions can be defined as rather short-lived, object-directed and high in intensity, in contrast to moods, and for the purpose of this paper are best described by the approach of appraisal theory (Moors, 2010; Moors et al., 2013). Typically, humans experience emotions more strongly when events are relevant to their current needs, aims, motives, values, norms, attachments, beliefs, or expectations (Frijda, 1986; Lazarus, 1991; Scherer, 2005). Furthermore, emotions can make experiences more memorable (Reisberg and Heuer, 1992). In this sense, emotions are functional, guiding us in adaptive responses to social relational opportunities and challenges (Fiske, 2002, 2010). For instance, although often perceived as negative, the primary function of shame, grief, guilt, and embarrassment is to establish, regulate, and maintain social relationships and social positions relative to others (Bastin et al., 2016, p. 457). Due to the specific underlying function of establishing, regulating and maintaining relationships, these emotions can be categorized as *social relational emotions*.

More recently, emotions with positive valences have also received scientific attention. These are awe (Keltner and Haidt, 2003; Shiota et al., 2007), admiration (Onu et al., 2016), gratitude (McCullough et al., 2001; Ma et al., 2017), compassion (Nussbaum, 1996), and moral elevation (Haidt, 2003; Pohling and Diessner, 2016). All of these emotions seem capable of boosting a sense of connection with others and can, therefore, be categorized as *positive social relational emotions*. For instance, Stellar et al. (2017) highlighted that emotions like awe, gratitude, and compassion are powerful proximal determinants of prosocial action. According to the authors, it is through prosocial tendencies, that these emotions (termed by the authors as *self-transcendent emotions*) bind individuals to others.

In this context, we highlight the potential of one specific positive emotion, kama muta, (Sanskrit: being moved by love; Seibt et al., 2017; Fiske et al., 2019; Zickfeld et al., 2019), which may be the most crucial social relational emotion in connectedness. Holding a new-born baby in your arm, surprisingly seeing a loved one again after a long time, or unexpectedly receiving a great kindness are typical example of moments in which people experience kama muta. In vernacular English, kama muta is often described as being *moved* or *touched*, while the elicitors may be called *heartwarming* (Fiske et al., 2019). The primary appraisal involved in kama muta is experiencing a sudden intensification of communal sharing. A number of studies suggest that kama muta has evolved (biologically and culturally) to regulate communal sharing relations (Seibt et al., 2017; Schubert et al., 2018). Communal sharing, one out of four relationships humans use to coordinate their social interactions, is the foundation of relationships in which people feel shared identity, are motivated by unity, share resources according to need and ability or signal and commit to being one by assimilating each other’s bodies (see: Relational Models Theory: Fiske, 1991, 1992, 2004). While kama muta is often evoked by the perception of a sudden intensification of a communal sharing relationship with another human being, the theory is not restricted to it. It explicitly suggests that people may feel kama muta when suddenly intensifying communal relationships with an animal, deity or even an abstract entity such as the earth or the cosmos. Therefore, kama muta is likely to play an important role in nature connectedness.

## The Role of Social Relational Emotions in Nature Experiences

In this section, we are raising awareness for the fact that aspects of social relational emotions have been associated with psychological inspired research on nature experiences for the last 50 years, but rarely investigated empirically. By pointing to some current empirical research in this field, we highlight the potential that social relational emotions offer for understanding the gateways to nature-connectedness and the associated outcome variables such as well-being.

Although not explicitly conceptualized as such, social relational emotions have long been recognized in the philosophical literature on environmental ethics. Naess (1977), who builds his theoretical considerations regarding deep ecology upon the ideas of Spinoza, views emotions to be fundamental to the complex inter-relationships that the natural world consists of. Early philosophically-inspired researchers looking into psychological aspects of connecting experiences in nature tended to ascribe emotional characteristics to nature experiences without separating them from other aspects of the experience. For instance, based on the concept of *peak experiences* by Maslow (1964), a study with a sample of 1,000 Americans showed that 82% of the participants indicated that they had experienced the beauty of nature in a deeply moving way (Wuthnow, 1978). A study by DeMares (2000) found similar emotional responses. According to the interview respondents, the encounter with cetaceans led to feeling connected with the animal and was sometimes described as a life-changing peak experiences. Expanding on the concept of peak experiences, the ecopsychologist Davis (1998) proposed the term *transpersonal experiences in nature*, which includes the experience of peace, joy, love, support, inspiration, and communion. According to the author, those are aspects of nonreligious spiritually. Later, Trigwell et al. (2014) identified such non-religious spirituality as a mediator of nature connectedness and eudaimonic well-being. Additionally, Marshall (2005) refers to *mystical experiences* in which people feel that the natural world evokes a sense of unity, knowledge, self-transcendence, eternity, light, and love. The notion of emotions as part of some form of spirituality is widely noted in the literature when trying to explain processes of nature connectedness. However, a more recently published paper by Lumber et al. (2017) investigated whether the nine values of the Biophilia Hypothesis, which represent a combination of different values and emotions, mediate nature-connectedness. Through two online surveys (*n* = 321) and one walking intervention (*n* = 72), they found that contact with nature, emotion, meaning, compassion, and beauty are pathways to improving nature connectedness. Another recent research project by Anderson et al. (2018) used daily diary methods to look at distinct emotions and their mediating role in nature experience for the outcome of well-being. In their first study, they found that awe experienced by 72 military veterans and 52 young people from underserved communities while white-water rafting, above other positive emotions measured, predicted increases in well-being, and reduction of stress-related symptoms after one week. In the second study, they showed that the nature experiences of 115 undergraduate students during their everyday lives led to more awe, which mediated the effect of nature experience on improvements in well-being. Additionally, gratitude and awe each mediated the effect of nature experience on daily life satisfaction.

In sum, research on social relational emotions and nature-connectedness presents itself as relativity vague and indicates that it includes a mix of social relational emotions, often labeled as *spiritual* aspects. Such a theoretical framing makes it challenging to scientifically investigate emotional processes. However, English-speakers’ descriptions of experiences as *deeply moving* or even *love* might represent instances of the kama muta emotion. Moreover, despite some weaknesses in the methodology, single empirical examples reveal that emotions in general, and compassion specifically, could be important for the sense of nature connectedness. Furthermore, the literature suggests that social relational emotions such as awe and gratitude play a direct role in well-being that result from contact to nature.

## Conclusion and Implications for Future Research

Understanding the emotions that facilitate and strengthen a sense of nature-connectedness has the potential to inform moves to increase human well-being and to foster pro-environmental attitudes and behavior. Social-relational emotions, especially kama muta, seem to be salient in experiences of connection with nature. This has five implications.

First, future research should investigate empirically, how, singly or in combination, of social relational emotions such as awe, compassion, gratitude, moral elevation, and kama muta effect connectedness to nature. Researching emotions and relationships is complex. This and the fact that we propose a new area of research invites multiple methodological approaches. While systematic approaches are required to explore the structure of the experienced phenomenon on the individual level, for instance, by applying the descriptive phenomenological method (Giorgi, 2009), systematic approaches on a larger scale are essential to allow for generalization of findings. For example, collecting data though the mobile experience sampling method (mESM) could address the fact that emotional states are brief experiences at particular moments in time. Applying mESM by using smartphone technology is a method of continuous recording of people’s daily life in real time to assess emotional states on a within and between level (Pejovic et al., 2016). In comparison to traditional self-report, the ESM method is less subject to biases introduced by recall and retrieval processes (Shiffman and Stone, 1998; Stone et al., 1998).

Second, it is important to recognize that every experience, including its underlying emotions, is culturally situated and culturally informed. Connectedness to nature is fostered by cultural models of *nature* (Wohlwill, 1983). This makes it essential to conduct research across cultures that consider how cultural conceptions of nature enable, limit, and shape social relational emotions. If theory should guide practice, future research in this area should accordingly be based on established and cross-culturally applicable theory-based emotions, such as kama muta, which is labeled with a Sanskrit term in order to avoid the confusions of vernacular lexemes (Fiske, 2019). Validated measures should be used to facilitate comparison among studies as well as the generalization of findings. For instance, for kama muta, there exists the KAMMUS-Two scale developed by Zickfeld et al. (2019) across a variety of contexts, in 19 countries and 15 languages. A well-established and valid questionnaire for gratitude is the Gratitude Questionnaire-6 (GQ-6) (McCullough et al., 2002), and for the emotion awe, the Awe Experience Scale (AWE-S) (Yaden et al., 2019).

Third, given Anderson et al.’s (2018) finding that experiencing the emotion of awe in nature predicts well-being, it will likely be fruitful to explore whether other social relational emotions mediate the effects of nature connectedness on well-being and pro-environmental attitudes and behavior. Positive emotions initiate a broader range of thoughts and actions than negative emotions do and have in general been identified as affording well-being and happiness (Diener and Seligman, 2004; Cohn et al., 2009). However, emotions like grief or guilt about injury to the environment or loss of nature may have substantial effects on people’s sense of responsibility for nature and may foster care for it. Thus, it would be particularly interesting to compare the effects of negatively perceived social relational emotions, such as grief or guilt, with positively perceived social relation emotions such as compassion, awe, or gratitude.

Fourth, despite the fact that current research emphasizes the benefits of direct engagement with nature, it simultaneously points to mounting evidence that physical contact with nature is decreasing (Rosa et al., 2018). In this context, Chirico et al. (2018) compared virtual exposure to nature and actual contact with nature. The results indicate that while browsing nature photographs or watching a nature documentary are likely to improve mood, getting outdoors, and connecting directly with nature was associated with better well-being benefits. Future research needs to address the question of how to maintain and adapt the possibilities for social relational emotions and human-nature connectedness to take place when technology is the medium to provide virtual exposure to nature. Studies in this area can be realized through experimental settings using virtual reality.

Finally, we have argued that social connectedness and connectedness to nature are underpinned by the same social relational emotions. However, do these emotions satisfy the human needs to relate in the same way or to the same degree when connecting to nature compared to connecting to other human beings? In other words, are there social relational emotions and qualities of relating or belonging that can only or more easily be enabled through experiences in nature, or through experiences with other humans? Future research should address such questions in order to provide guidance to practical application such as psychological or environmental therapy for attachment disorders, depression, anxiety, and other conditions.

## Final Remarks

**Image 1:**
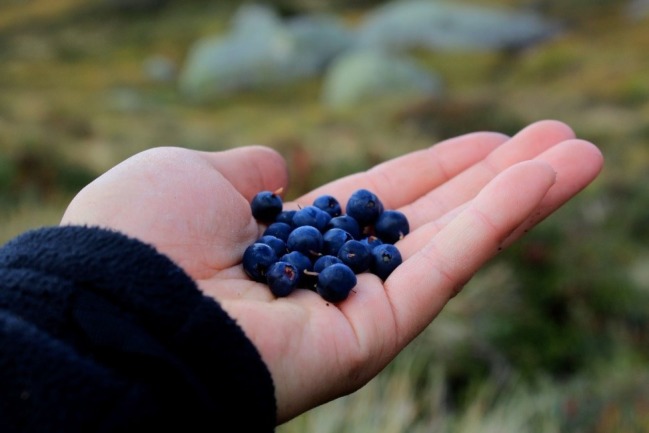
Connecting to nature. Norway, 2018. Picture taking by Joanna Stüber.

Above we quoted John Muir, “When one tugs at a single thing in nature, he finds it attached to the rest of the world”. Grasping any aspect of nature affords possibilities to ameliorate the well-being of individuals, communities, and the environment – because as we grasp a bit of nature, in turn nature tugs at our heartstrings (Image 1).

## Author Contributions

EP conceptualized and wrote the first draft. AF and TS contributed with advice. All authors revised the final manuscript.

### Conflict of Interest

The authors declare that the research was conducted in the absence of any commercial or financial relationships that could be construed as a potential conflict of interest.
